# Supplementary material for: Non-monotonic changes in Asian Water Towers’ streamflow at increasing warming levels
Source: Nat Commun. 2023 Mar 1;14:1176. doi: 10.1038/s41467-023-36804-6 (PMC9977870; doi:10.1038/s41467-023-36804-6)
Supplement: Supplementary file 1 — Supplementary Information [file 41467_2023_36804_MOESM1_ESM.pdf]

Supplementary Information for

Non-monotonic Changes in Asian Water Towers' streamflow at increasing Warming Levels

Content of the SI:

- Supplementary Methods
- Supplementary Figures 1-13
- Supplementary Tables 1-11

Supplementary Methods

Calculation of potential evapotranspiration (PET) based on the CMIP6 model output

We adopt the method developed by Van Pelt et al. (2009)[1] to calculate PET based on daily temperature as follows:

$$PET=[1+\alpha_0(T-\bar{T}_0)]\cdot\overline{PET}_0 \tag{1}$$

where $\bar{T}_0$ and $\overline{PET}_0$ are the daily mean temperature (in °C) and potential evapotranspiration (in mm day^{-1}) for the calendar month during the period 1961-2001 (provided by the WFD), respectively; T is the daily temperature from the CMIP6 model output (in °C); α_0 is determined for each calendar month by regressing the WFD-based PET to daily temperature over each grid.

Model physics of the modified THREW

A modified version of the Tsinghua Representative Elementary Watershed (THREW) is adopted in this study. The modified THREW that couples the modules of snowpack and glacier evolution is adopted in this study. The snow water equivalent (SWE) and snow cover area (SCA) is updated based on the following equations (also see ref 2, 3, 4)

$$M_s=\begin{cases}DDF_s\cdot(T-T_{s0}) & \text{for } T>T_{s0}\\ 0 & \text{for } T\leq T_{s0}\end{cases} \tag{2}$$

$$P_s=\begin{cases}0 & for\ T>T_s\\ P & for\ T\leq T_s\end{cases} \tag{3}$$

$$\frac{dSWE}{dt}=P_s-M_s \tag{4}$$

$$SCA=A\cdot\left(\frac{SWE}{WMAX}\right)^{LL} \tag{5}$$

where M_s is the snowmelt amount from the snowpack (in mm); T is air temperature (in °C); T_{s0} refers to the temperature threshold above which snow starts to melt (in °C); DDF_s is the degree-day factor for snow, representing the snow melt rate (in mm °C^{-1} day^{-1}); P_s is the amount of snow (in mm), and is determined

by a temperature threshold T_s (in °C); A, $WMAX$ (in mm) and LL are parameters used in the snow cover depletion curve to calculate SCA (in km^2) according to SWE (in mm).

To simulate the evolution of glaciers, an algorithm proposed by Luo et al. (2013)[5] is adopted to measure the changes of glacier mass. The simulated glacier mass balance components include mass accumulation, melting of ice and snow, and refreezing of these melt water. The ice and snow melt is simulated using the degree-day factor approach introduced by Hock et al. (2005)[6]. Based on the entire glacier mass balance, the glacier volume is calculated. The glacier area is updated using the glacier volume-area scaling relation introduced by Chen and Ohmura (1990)[7] (Equation 12).

The initial glacier extent within China is determined based on the First Chinese Glacier Inventory (CGI-1)[8]. For the regions outside China, we derive the initial glacier extent based on the Randolph Glacier Inventory Version 6.0 (RGI6.0)[9] and the estimated annual glacier changes rate (0.04-1.57%/yr) (see ref [10, 11, 12] for details). The Second Chinese Glacier Inventory (SCGI) and RGI6.0 are used to validate the capability of the THREW model in capturing the dynamical changes of the glacier extent. The initial glacier thickness is computed based on an area-volume scaling relationship, and is constrained based on Millan et al. (2022)[13].

In this study, each Representative Elementary Watershed (REW) is divided into different elevation bands with an equal interval of 200 meters. This is used to consider the spatial heterogeneity in meteorological forcing. The glacier regions within an elevation band are regarded as a glacier unit (GREW). For each GREW, the glacier change is simulated using the glacier module similar to the one that is successfully incorporated into the SWAT model by Luo et al. (2013)[5]. The equations are as follows.

$$P_{sg}=\begin{cases}0 & for\ T>T_{sg}\\ P & for\ T\leq T_{sg}\end{cases} \tag{6}$$

$$M_{sg}=\begin{cases}DDF_s\cdot(T-T_{s0}) & \text{for } T>T_{s0}\\ 0 & \text{for } T\leq T_{s0}\end{cases} \tag{7}$$

$$Eng=\beta\cdot Ws \tag{8}$$

$$\frac{dWs}{dt}=P_{sg}-M_{sg}-E_{ng} \quad (9)$$

$$M_g=\begin{cases} 0 & \text{for } Ws>0 \\ DDF_g\cdot(T-T_{g0}) & \text{for } Ws=0,\ T>T_{g0} \\ 0 & \text{for } Ws=0,\ T\leqslant T_{g0} \end{cases} \quad (10)$$

$$\frac{dH_g}{dt}=P_{sg}-M_{sg}-M_g \quad (11)$$

$$A_g=\left(\frac{V_g}{m_g}\right)^{\frac{1}{n_g}}=\left(\frac{H_g\cdot 10^{-3}A_{g0}}{m_g}\right)^{\frac{1}{n_g}} \quad (12)$$

where Equations (6) and (7) simulate the processes of snowfall and snowmelt for the glacier areas, similar to Equations (2) and (3). Eng is the amount of snow transferring to ice (in mm), which is assumed as a ratio of water equivalent of snow over ice, β is a constant accumulation coefficient, and Ws is the water equivalent of snow over ice in glacier cover areas (in mm). Equation (9) is the mass balance of snow over ice (M_g, in mm). Ice melt is calculated by the degree-day method using different degree-day factors (DDF_g, in mm °C^{-1} day^{-1}) from snow, and the melting process occurs only when there is no snow over ice. H_g is the thickness of a glacier (in mm), and is updated by the balance Equation (11). The area of glacier (A_g, in km^2) is updated by the volume-area scaling relation using the two parameters m_g and n_g in Equation (12). The glacier volume (V_g, in km^3) is calculated using the initial glacier area (A_{g0}) for each simulation time step.

There are 19 parameters in THREW, as listed in Supplementary Table 7. In this study, the parameters related to snow and glacier are separately calibrated against observational snow and glacier datasets. In the study, we just calibrate the parameters related to the melting rate ($DDF_g$and DDF_s) and volume-area scale (i.e., A, $WMAX$, LL, n_g, m_g). Other parameters related to snow and glacier use the suggested values in the literature. All the temperature thresholds are assumed as 0 °C, and the ratio of snow transferring to ice is assumed 0.5. Due to the important role of the melt water for the Indus, thresholds related to temperature are automatically calibrated based on PySOT. Calibrated values of temperature thresholds are 4.906 °C, -2.748 °C, and 4.164 °C for T_{s0}, T_{sg} and T_s respectively.

Sensitivity analyses of the glacier module parameters

Glaciers play an important role in hydrological processes over the Tibetan Plateau. To assess the influence of the glacier on the resulting runoff change, we performed a series of sensitivity experiments for the Indus. This is because the Indus has the largest contribution of glacier runoff to total runoff among all seven river basins. Given that the volume-area scaling technique heavily depends on the area of glaciers, a straightforward method, also used by Frey et al. (2014)[14], is adopted to assess the sensitivity of hydrological response to glacier changes. The initial glacier extent was set based on the RGI6.0 (i.e. 19058 km^2 glacier area in the year 2012) and a calibrated parameter, the annual glacier changes rate (δ). The range (-0.04 ~ -1.57%/yr) for δ is determined by referring to previous studies[10, 11, 12]. The calibrated value of δ is -1.30%/yr for the Indus. This means that the initial glacier area within the Indus is 30559 km^2 at the start of the historical simulation. Considering the glacier evolution, δ of -1.57%/yr and of -0.04%/yr is assumed as the upper and lower bound for uncertainty in the input glacier area, respectively. Besides, the calibrated δ (-1.3%/yr) is changed by ±5%. Another important parameter influencing the results of ice-melt amount is degree-day factors (Equation 10). Similar to the parameter δ, a set of modified degree-day factors (with the calibrated value modified by ±10% and ±5%, respectively) are used. The influence of the δ and degree-day factor on changes in river flows under different warming scenarios are shown in Supplementary Table 11. The non-monotonic changes in river flows are still maintained with varying parameters of the glacier module. We expect our conclusions would also hold with varying parameters of the glacier module for other basins as well, since the contributions of glacier runoff to total runoff are much smaller over other basins than the Indus.

Supplementary Figures

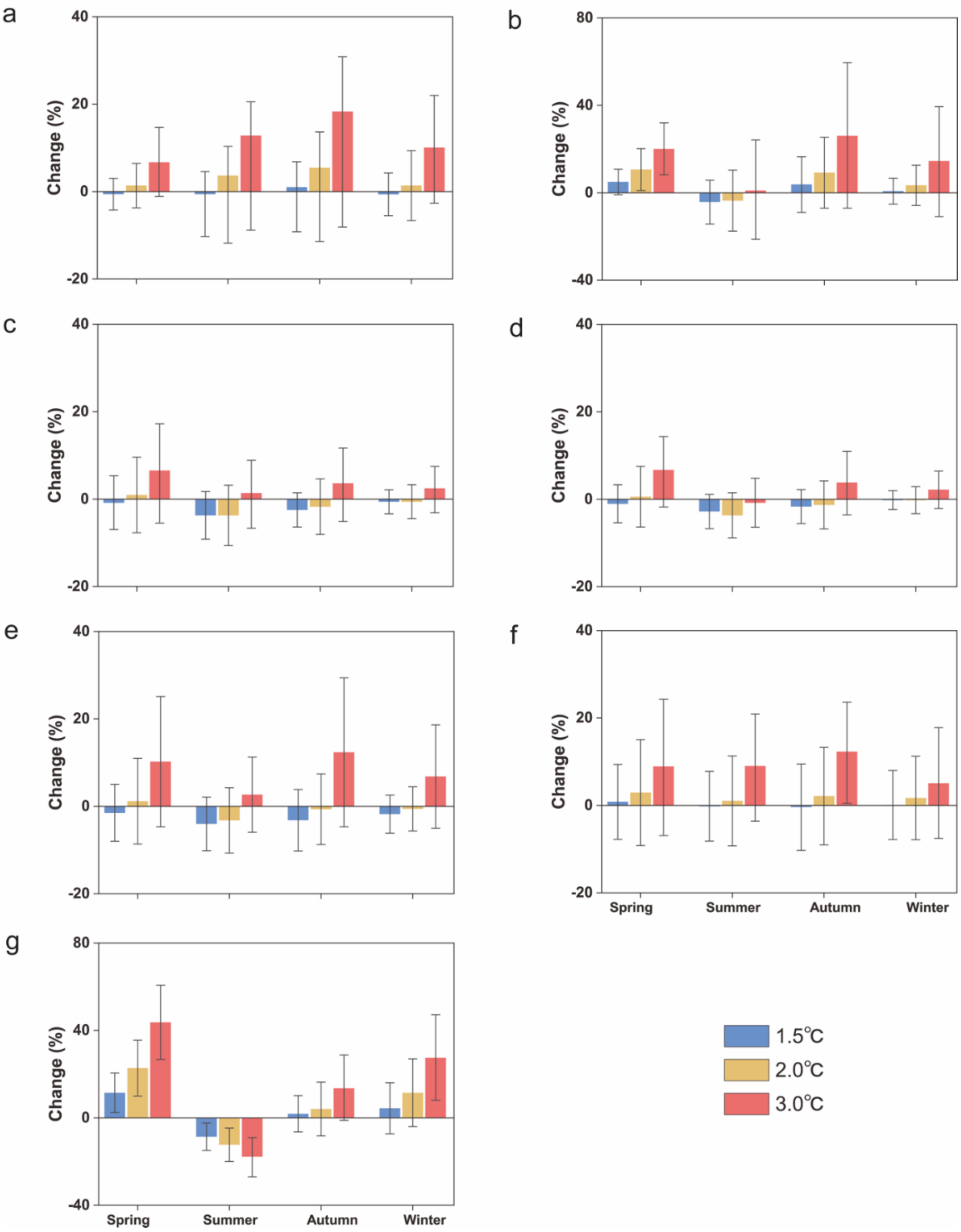

Supplementary Fig. 1 Relative changes of seasonal river flow at the warming levels of 1.5 °C, 2.0 °C and 3.0 °C for the seven river basins. a Yellow; **b** Yangtze; **c** Mekong; **d** Salween; **e** Brahmaputra; **f** Ganges; **g** Indus. Color bars represent the ensemble mean from the hydrological simulations driven by the 22 CMIP6 models, while error bars represent one standard deviation. The four seasons are defined as spring (March-May), summer (June-August), autumn (September-October), and winter (November to February of the following year).

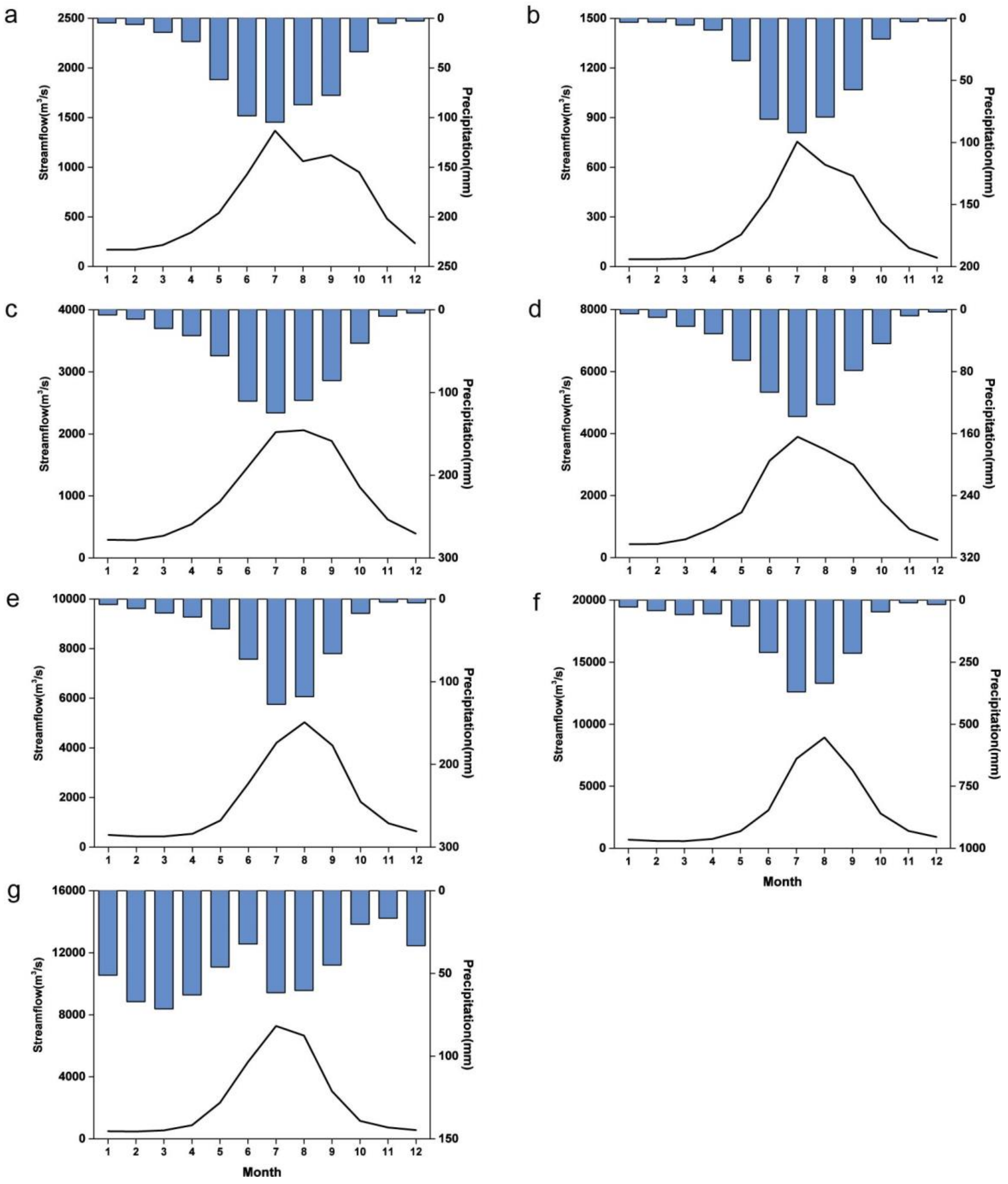

Supplementary Fig. 2 Monthly streamflow and precipitation of the seven river basins during the historical period. **a** Yellow; **b** Yangtze; **c** Mekong; **d** Salween; **e** Brahmaputra; **f** Ganges; **g** Indus. Streamflow and precipitation (from the WATCH Forcing Dataset) are calculated within the observed period of streamflow for each river basin (see Supplementary Table 1 for details).

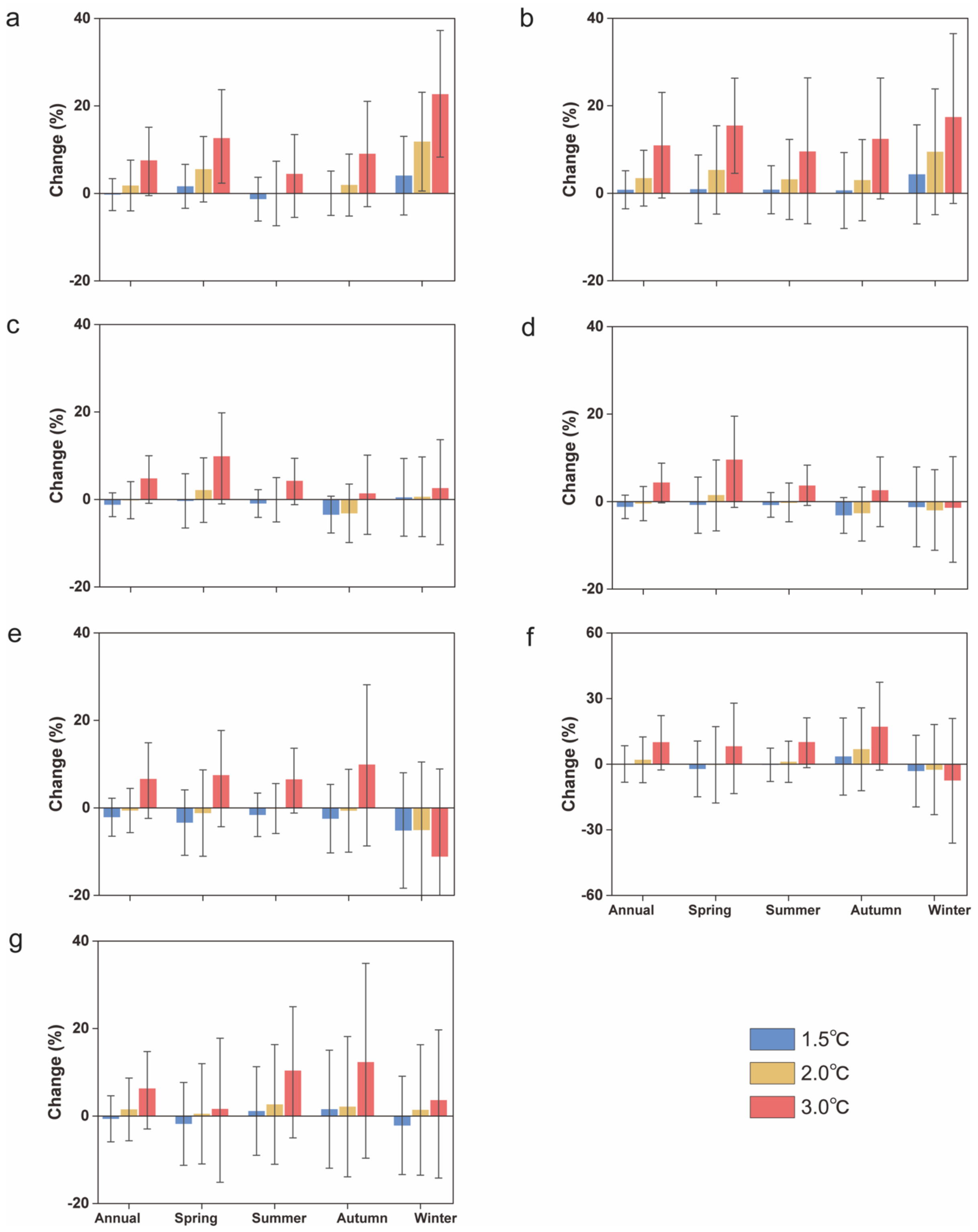

Supplementary Fig. 3 Relative changes of precipitation at the warming levels of 1.5 °C, 2.0 °C and 3.0 °C for the seven river basins. a Yellow; **b** Yangtze; **c** Mekong; **d** Salween; **e** Brahmaputra; **f** Ganges; **g** Indus. Color bars represent the ensemble mean from 22 CMIP6 models, while error bars represent one standard deviation

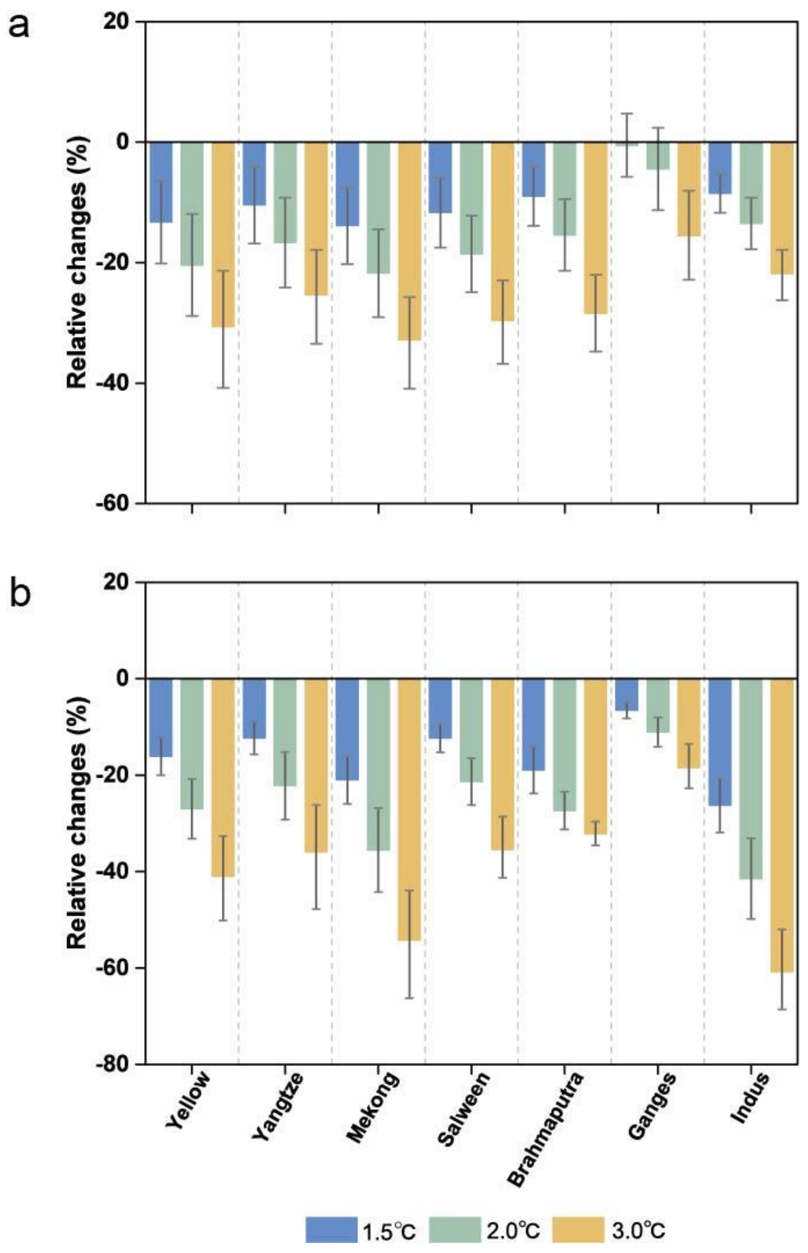

Supplementary Fig. 4 Relative changes of snow cover and glacier area at the warming levels of 1.5 °C, 2.0 °C and 3.0 °C for the seven river basins. **a** percentage of snow cover (PSC); **b** glacier area. Color bars represent the ensemble mean from the hydrological simulations driven by the 22 CMIP6 models, while error bars represent one standard deviation.

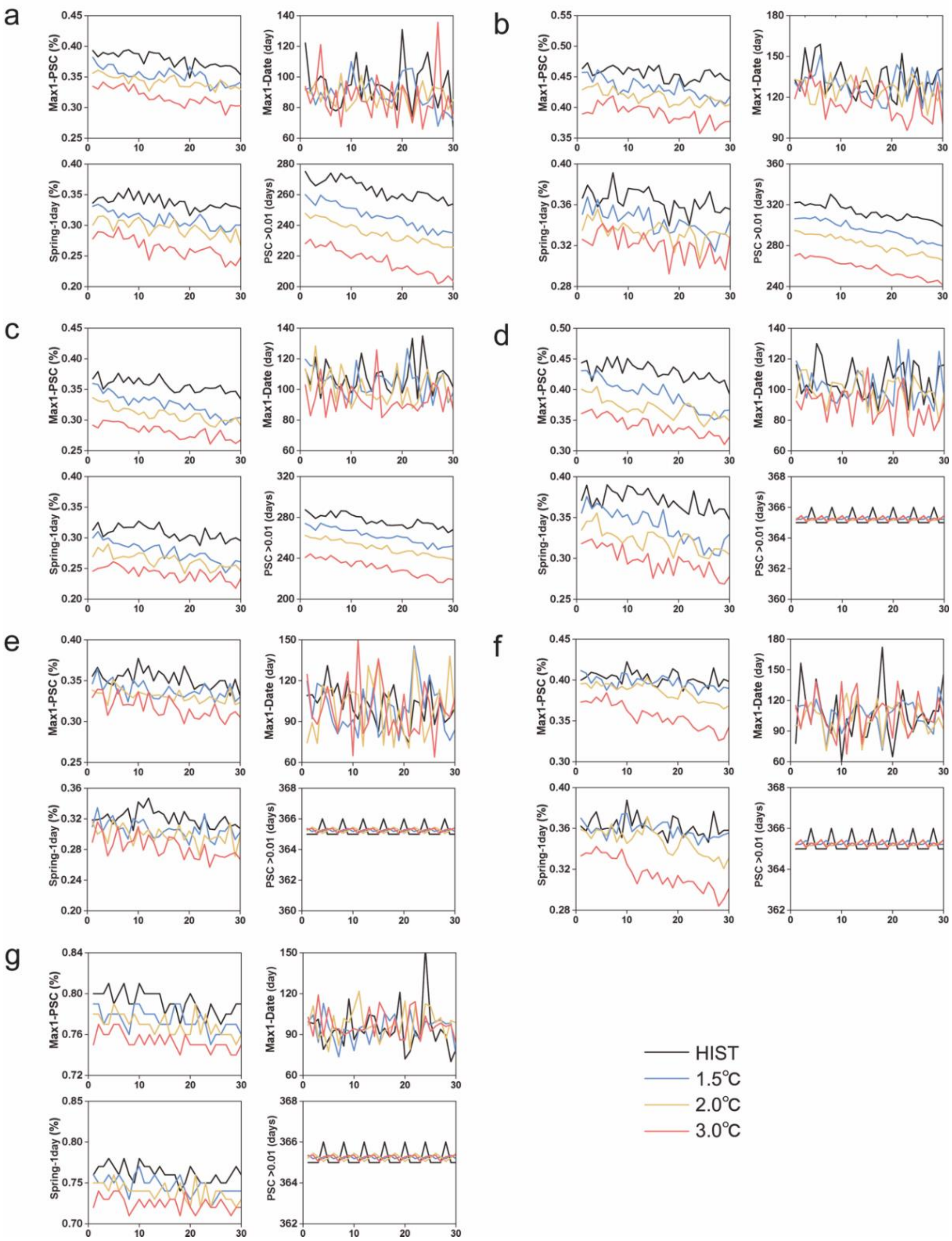

Supplementary Fig. 5. Variation of the percentage of snow cover (PSC) during the historical period (HIST, 1985-2014) and at the global warming levels of 1.5 °C, 2.0 °C and 3.0 °C for the seven river basins. a Yellow; **b** Yangtze; **c** Mekong; **d** Salween; **e** Brahmaputra; **f** Ganges; **g** Indus. Max1-PSC: annual 1-day maximum PSC; Max1-Date: Julian date of the annual 1-day maximum PSC; Spring-1day: 1-day maximum PSC in spring. Note that each solid line shows the ensemble mean during a 30-year running period. The grids with PSC exceeding 0.01 are considered.

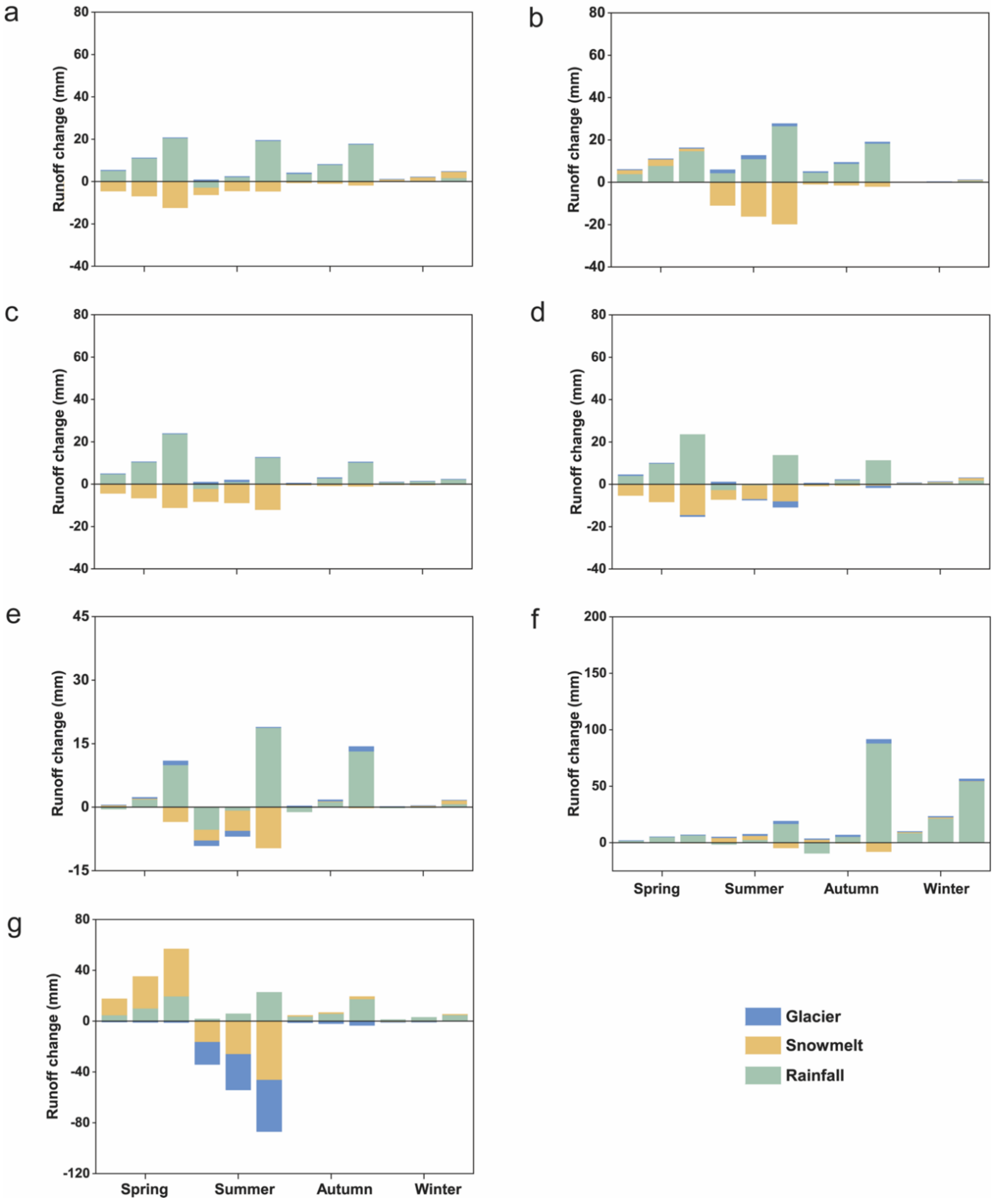

Supplementary Fig. 6 Relative changes in mean seasonal rainfall runoff, snowmelt runoff, and glacier runoff at the warming levels of 1.5 °C, 2.0 °C and 3.0 °C for the seven river basins. **a** Yellow; **b** Yangtze; **c** Mekong; **d** Salween; **e** Brahmaputra; **f** Ganges; **g** Indus. Three columns from left to right in each season represent the global warming levels of 1.5 °C, 2.0 °C and 3.0 °C, respectively.

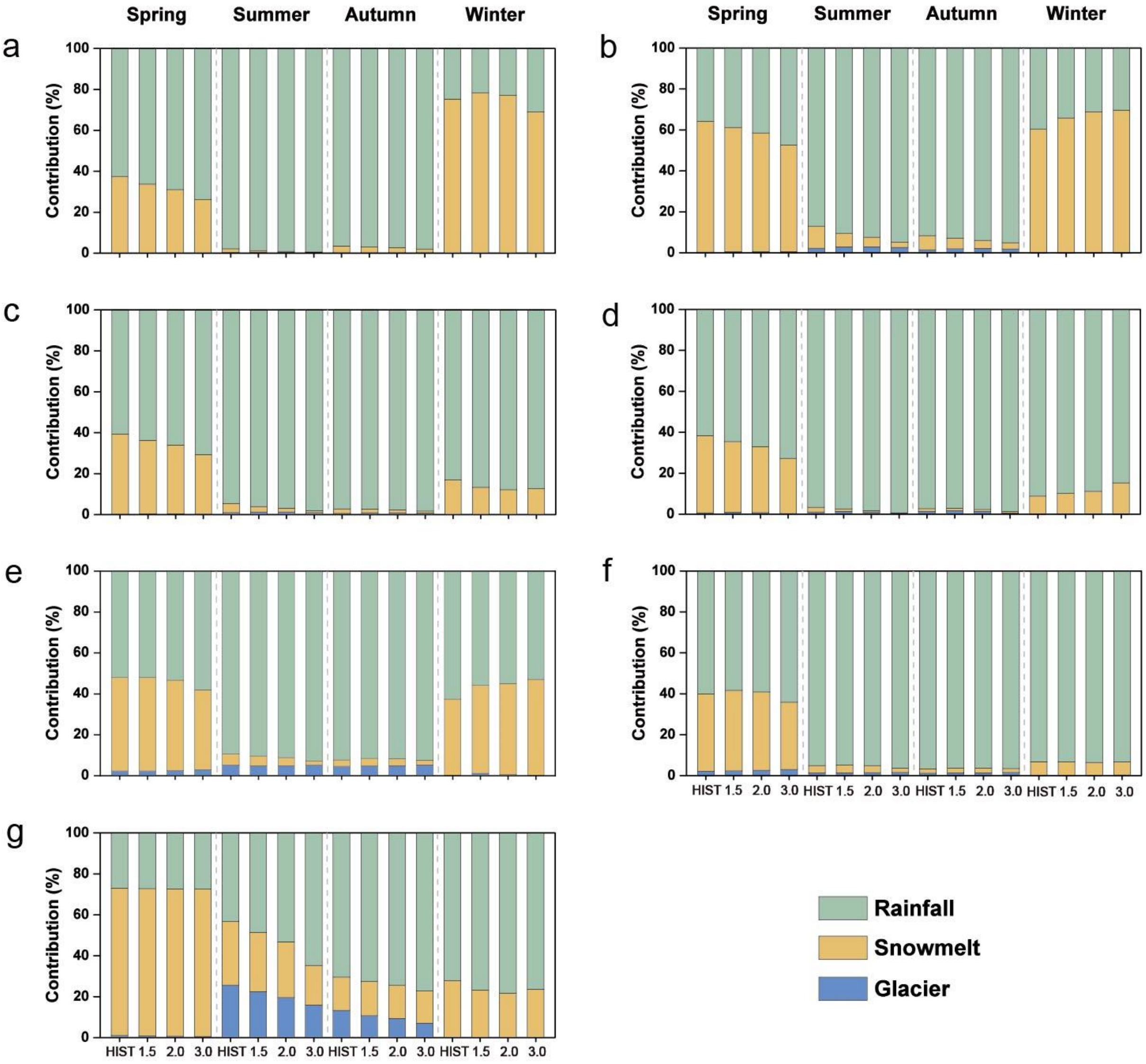

Supplementary Fig. 7 Relative contribution of different runoff components to seasonal runoff at the warming levels of 1.5 °C, 2.0 °C and 3.0 °C for the seven river basins. **a** Yellow; **b** Yangtze; **c** Mekong; **d** Salween; **e** Brahmaputra; **f** Ganges; **g** Indus. The first column shows the contribution during the historical period (HIST), while the second to the fourth columns show the contribution at the global warming levels of 1.5 °C, 2.0 °C and 3.0 °C, respectively.

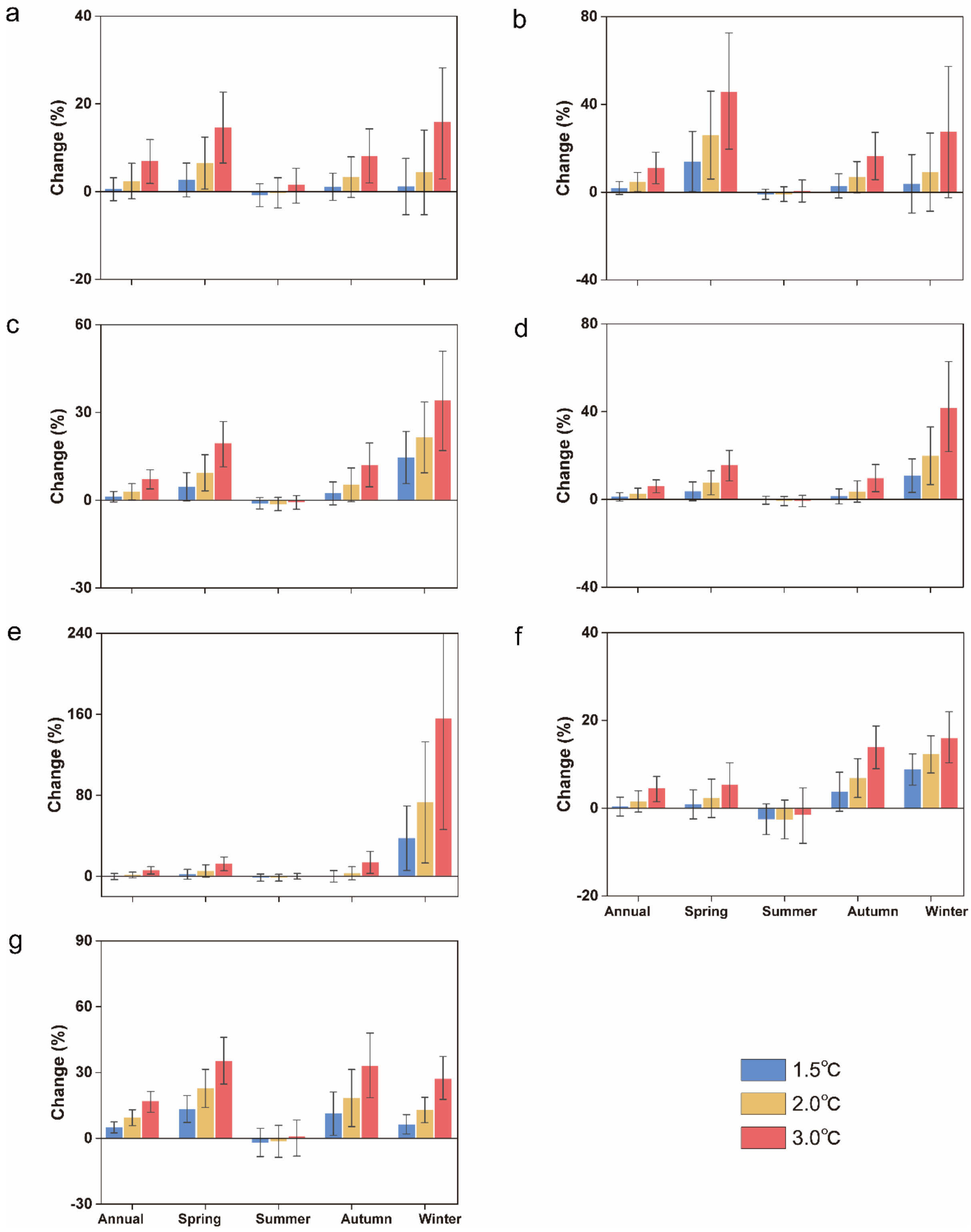

Supplementary Fig. 8 Relative changes of evapotranspiration at the warming levels of 1.5 °C, 2.0 °C and 3.0 °C for the seven river basins. a Yellow; **b** Yangtze; **c** Mekong; **d** Salween; **e** Brahmaputra; **f** Ganges; **g** Indus. Color bars represent the ensemble mean from the hydrological simulations driven by the 22 CMIP6 models, while error bars represent one standard deviation.

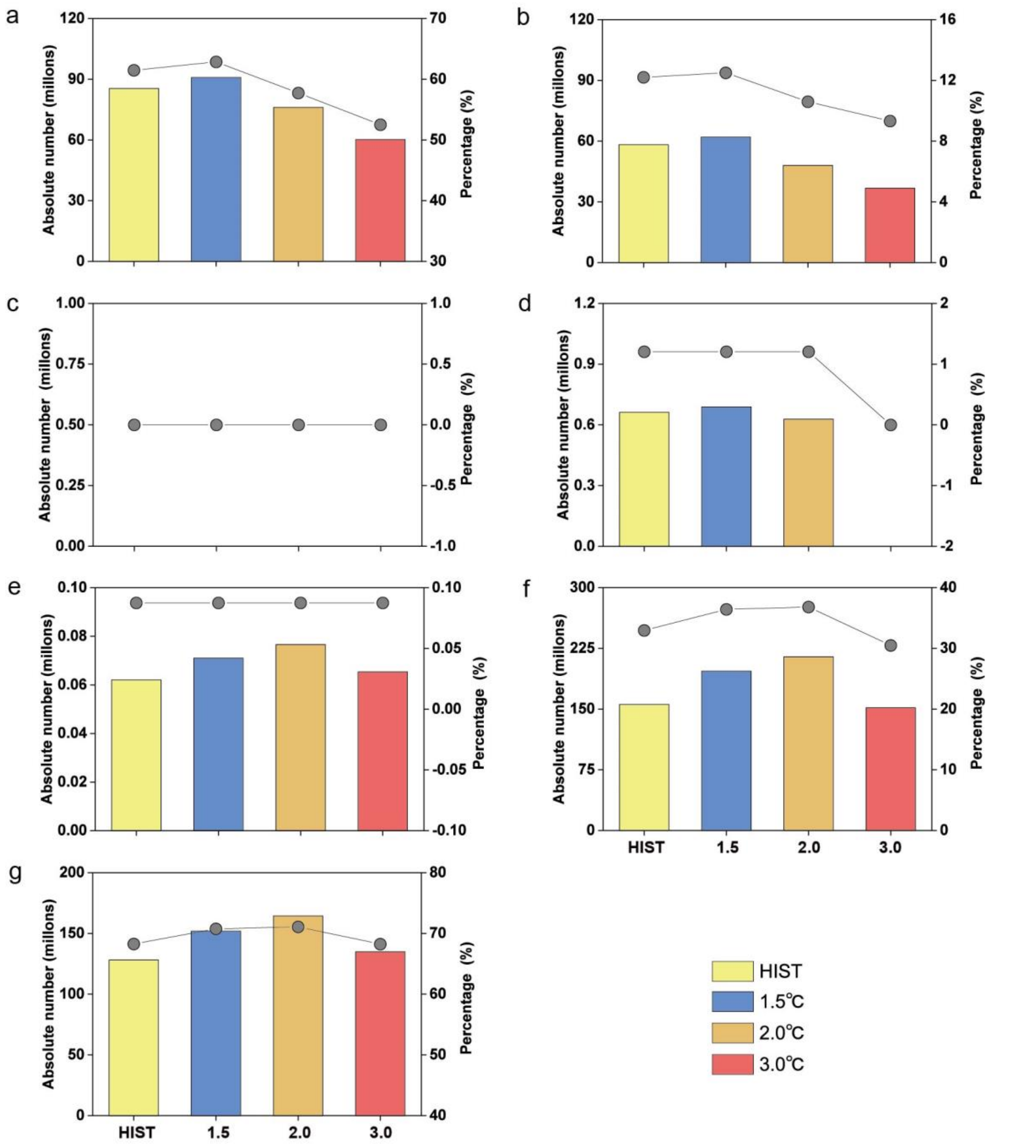

Supplementary Fig. 9 The absolute number and percentage of people under water scarcity at the global warming levels of 1.5 °C, 2.0 °C and 3.0 °C and during the historical period (HIST) for the seven river basins. a Yellow; **b** Yangtze; **c** Mekong; **d** Salween; **e** Brahmaputra; **f** Ganges; **g** Indus. Water scarcity conditions are assessed based on the water scarcity index. The grey points are the percentage of the population under water scarcity for the whole basin at different global warming levels. Note that the population under water scarcity for the Mekong (c) is zero.

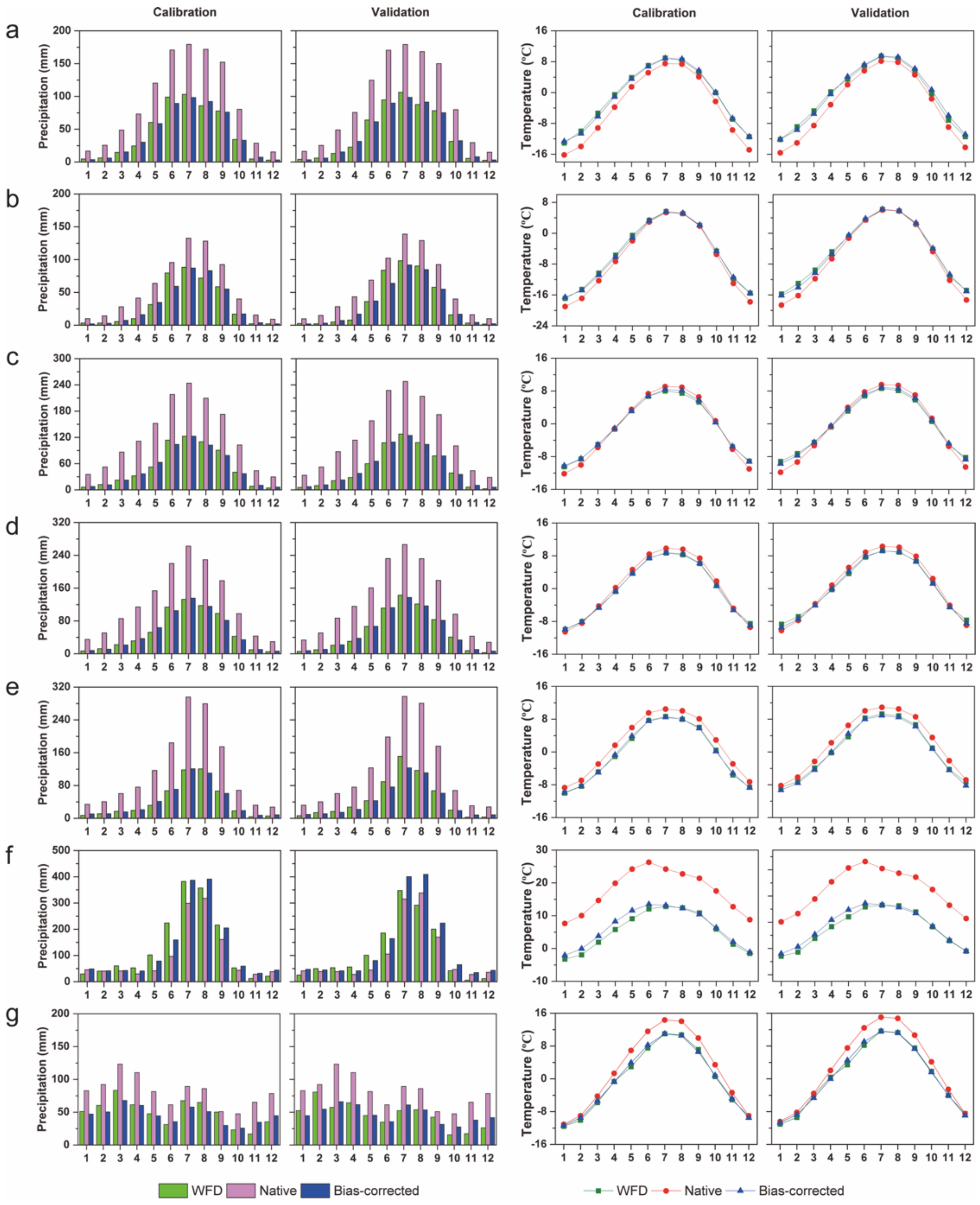

Supplementary Fig. 10 Seasonal cycles of precipitation and temperature calculated from the WATCH Forcing Dataset (WFD), the ensemble mean of 22 native and bias-corrected CMIP6 model output for the calibration (1980-2000) and validation (2001-2014) period. a Yellow; **b** Yangtze; **c** Mekong; **d** Salween; **e** Brahmaputra; **f** Ganges; **g** Indus. The left two columns represent precipitation, while the right two are temperature.

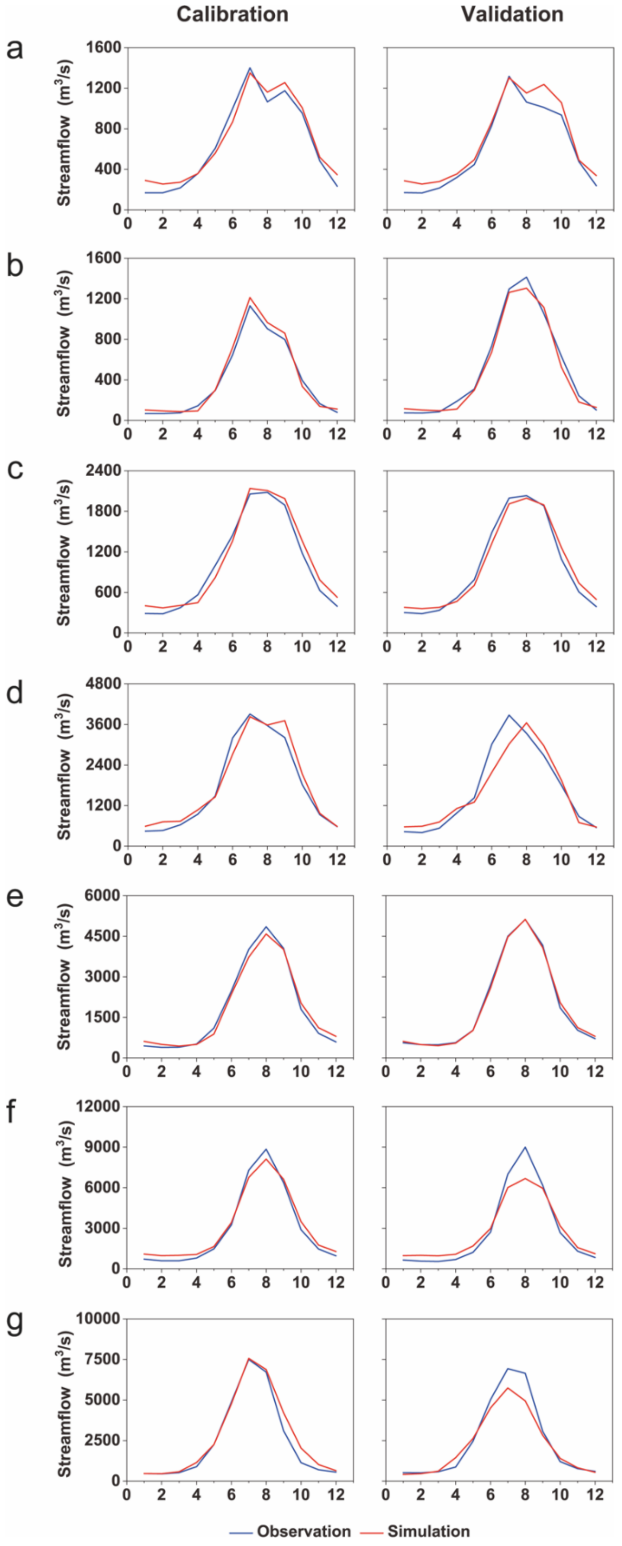

Supplementary Fig. 11 Comparisons of simulated (in red) and observed (in blue) monthly streamflow for the seven river basins. a Yellow; **b** Yangtze; **c** Mekong; **d** Salween; **e** Brahmaputra; **f** Ganges; **g** Indus. The left panels show simulated streamflow during the calibration period, while the right panels show results during the validation period.

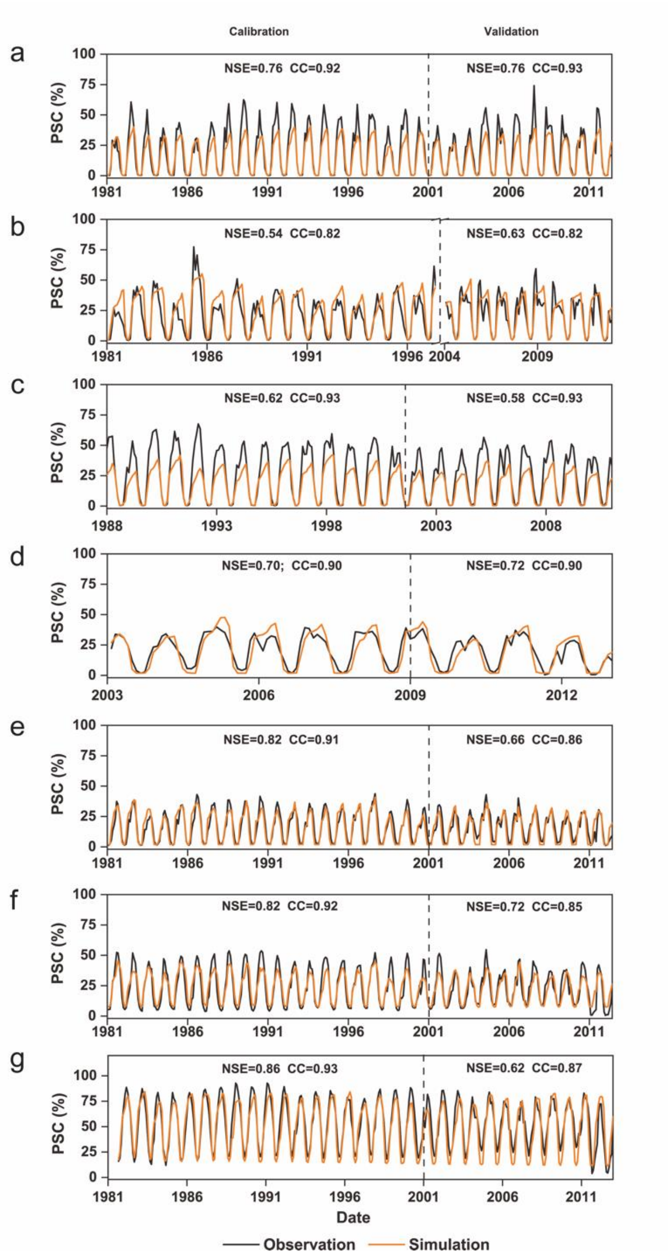

Supplementary Fig. 12 The observed (black line) and simulated (orange line) monthly percentage of snow cover (PSC) for the seven river basins. a Yellow; **b** Yangtze; **c** Mekong; **d** Salween; **e** Brahmaputra; **f** Ganges; **g** Indus. The observed snow coverage is provided by the long-term TP daily 5-km cloud-free snow cover extent dataset[15]. The Nash Sutcliffe efficiency coefficient (NSE) and correlation coefficients (CC) values are also shown in the figure.

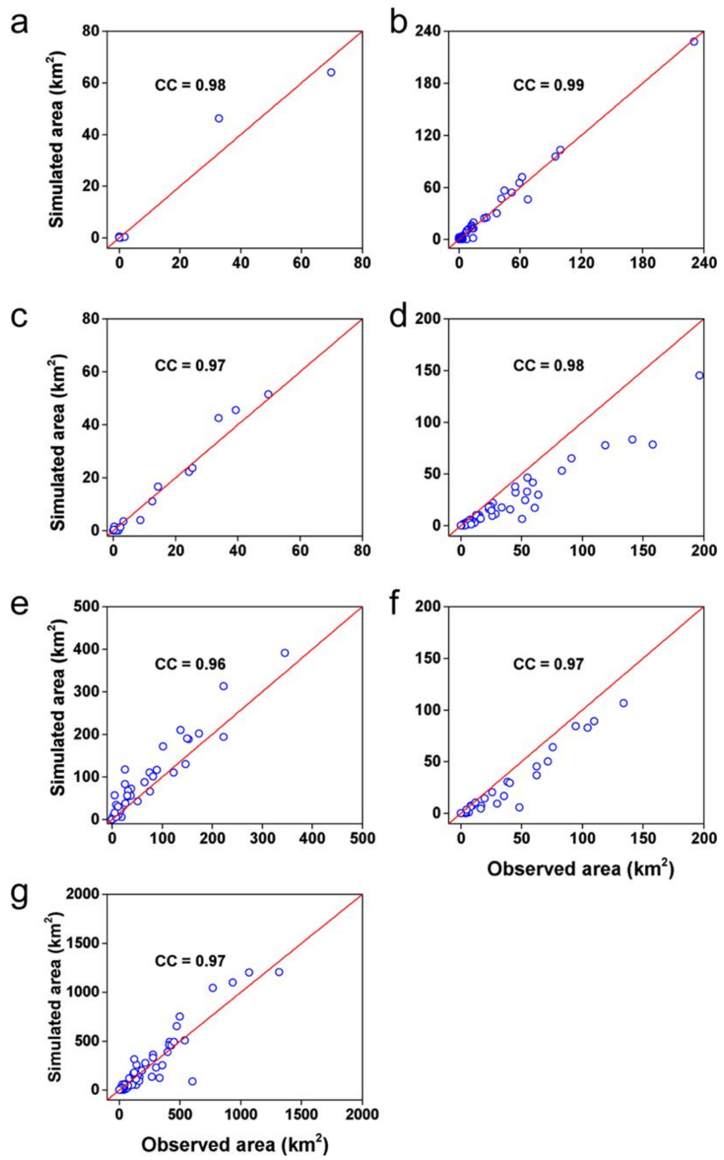

Supplementary Fig. 13 The correlation coefficients (CC) of the observed and simulated glacier coverage for the REW over the seven river basins. a Yellow; **b** Yangtze; **c** Mekong; **d** Salween; **e** Brahmaputra; **f** Ganges; **g** Indus. The observed glacier coverage is obtained from the SCGI and RGI6.0.

Supplementary Tables

Supplementary Table 1. The main characteristics of the seven river basins over Tibetan Plateau .

Basin	Yellow	Yangtze	Mekong	Salween	Brahmaputra	Ganges	Indus
Station	Tangnaihai	Zhimenda	Jiuzhou	Daojieba	Nuxia	Devghat/Chisapani	Besham
Drainage area (km^2)	121,000	137,700	88,051	110,224	205,072	80,541	164,867
Altitude range (minimum/mean/maximum) (m)	2656 /4124 /6253	3516 /4759 /6575	1243 /4323 /6385	647 /4486 /6891	2914 /4875 /7296	129 /3180 /8143	361 /4453 /8483
Dominant land covers (%)	Forest 4.5<br>Grassland 93.5<br>Bare 1.1	Grassland 92.6<br>Bare 5.8<br>Snow and ice 1.3	Grassland 78.4<br>Forest 17.8<br>Bare area 2.9	Forest 14.8<br>Grassland 76.3<br>Snow and Ice 4.0	Grassland 81.4<br>Forest 7.8<br>Snow and Ice 5.0	Forest 38.6<br>Grassland 27.7<br>Cropland 33.2	Forest 5.2<br>Grassland 42.8<br>Snow and Ice 46.5
Glacier area (km^2)	106	989	221	1793	2632	2854	19058
Observational period of daily streamflow	1980-2012	1980-1997, 2004-2012	1988-2010	2003-2012	1980-2012	1980-2012	1980-2012
Streamflow (m^3/s)	631	441	998	1729	1858	2902	2440
Annual Mean temperature (°C)	-1.1	-5.1	-0.5	0.46	-0.1	4.9	0.18
Annual Mean precipitation (mm)	518	386	609	627	503	1493	575
Runoff ratio	0.32	0.26	0.59	0.79	0.62	0.76	0.82

Note: Streamflow is calculated for the period of streamflow observations; Annual temperature and precipitation are based on the WATCH Forcing Data (WFD); Note that the precipitation in Indus is adjusted by multiplying the WFD by 1.61. Land covers are obtained from Global Land Cover 2000 map by the EC Joint Research Centre (http://bioval.jrc.ec.europa.eu/products/glc2000/glc2000.php).

Supplementary Table 2. Data from global and regional datasets used for hydrological models in this study.

Dataset	Source/Name	Description	Reference and/or Website for download
Climate	WATCH Forcing Data (WFD)	Daily meteorological forcing data at 0.5 arc degree grid global dataset	https://rda.ucar.edu/datasets/[16]
Topography	MERIT DEM	Developed by removing multiple error components from the SRTM3[17] and AW3D-30m DEMs[18]	Yamazaki et al. (2014)[19] http://hydro.iis.u-tokyo.ac.jp/~yamadai/MERIT_DEM/
Land use / cover	Daily normalized difference vegetation index (NDVI)	Daily NDVI values at 0.5 arc degree grid derived from Advanced Very High Resolution Radiometer (AVHRR) sensors	Vermote et al. (2019a)[20] https://www.ncei.noaa.gov/products/climate-data-records/normalized-difference-vegetation-index
Land use / cover	Leaf Area Index (LAI)	Daily product on a 0.05° by 0.05° grid from 1981 to present derived from Advanced Very High Resolution Radiometer (AVHRR) sensors	Vermote et al. (2019b)[21] https://www.ncei.noaa.gov/products/climate-data-records/leaf-area-index-and-fapar
Snow	Long-term TP daily 5-km cloud-free snow cover extent record (TPSCE)	Daily 5-km cloud-free snow cover extent record derived from AVHRR surface reflectance CDR[22]	Chen et al. (2018)[15]
Glacier distribution	The First Chinese Glacier Inventory (CGI-1)	Clear and concise overview and scientific assessment of the glaciers in China	Shi et al. (2009)[8]
Glacier distribution	The Second Chinese Glacier Inventory (SCGI)	Clear and concise overview and scientific assessment of the glaciers in China	Guo et al. (2015)[23]
Glacier distribution	The Randolph Glacier Inventory 6.0 (RGI6.0)	The Randolph Glacier Inventory is a global inventory of glacier outlines. It is supplemental to the Global Land Ice Measurements from Space initiative (GLIMS)	RGI Consortium (2017)[9]
Ice Thickness	Ice velocity and thickness of the world's glaciers	The datasets present a comprehensive high-resolution mapping of ice velocity and thickness of more than 98% of the world's glacier	Millan et al. (2022)[13]

Dataset	Source/Name	Description	Reference and/or Website for download
Glacier mass balance	Glacier mass balance data	The datasets provided standardized observations on changes in mass, volume, area and length of glaciers over time	World Glacier Monitoring Service (WGMS, 2021)[24] and Hugonnet et al. (2021)[25]
Soil	Global high-resolution data set of soil hydraulic and thermal parameters	Optimal soil water retention parameters obtained from ensemble pedotransfer functions	http://globalchange.bnu.edu.cn/research, Dai et al. (2019)[26]

Supplementary Table 3. Calibrated and validated results of the THREW model.

		Yellow	Yangtze	Mekong	Salween	Brahmaputra	Ganges	Indus
Number of REWs		215	178	54	355	63	147	74
Average area of REW (km^2)		657	792	1677	310	2971	547	2227
Calibration period		1981-2000	1981-1997	1988-2000	2003-2008	1981-2000	1981-2000	1981-2000
Streamflow (daily/monthly)	NSE	0.80/0.86	0.79/0.86	0.86/0.93	0.76/0.92	0.83/0.88	0.87/0.95	0.79/0.83
	PBIAS (%)	5.2/5.2	5.3/5.3	4.4/4.4	4.3/4.4	-0.1/-0.1	5.4/5.5	9.7/9.8
	CC	0.90/0.93	0.90/0.93	0.93/0.97	0.88/0.96	0.91/0.94	0.93/0.98	0.90/0.92
Snow cover (monthly)	NSE	0.76	0.54	0.62	0.70	0.82	0.82	0.86
	CC	0.92	0.82	0.93	0.90	0.91	0.92	0.93
Validation period		2001-2012	2001-2012	2001-2010	2009-2012	2001-2012	2001-2012	2001-2012
Streamflow (daily/monthly)	NSE	0.66/0.70	0.71/0.75	0.89/0.94	0.68/0.83	0.57/0.59	0.81/0.89	0.86/0.89
	PBIAS (%)	12.8/12.8	-4.7/-4.7	1.6/1.6	-3.0/-3.0	0.6/0.6	-0.8/-0.6	-9.8/-9.7
	CC	0.83/0.86	0.86/0.88	0.94/0.97	0.83/0.91	0.81/0.82	0.92/0.97	0.95/0.97
Snow cover (monthly)	NSE	0.76	0.63	0.58	0.72	0.66	0.72	0.62
	CC	0.93	0.82	0.93	0.90	0.86	0.85	0.87
Glacier area	PBIAS (%)	5.39	-1.89	2.05	-42.20	31.6	-30.4	-1.82
	CC	0.98	0.99	0.97	0.98	0.96	0.97	0.97

Here the Nash Sutcliffe efficiency coefficient (NSE), correlation coefficient (CC) and percent bias (PBIAS) are used to evaluate the model performance.

Supplementary Table 4. Details of the 22 CMIP6 climate models used in this study.

Model Name	Modeling Center	Realization	Resolution (Lon×Lat)
ACCESS-CM2	Commonwealth Scientific and Industrial Research Organisation, Australia	r1i1p1f1	1.875°×1.25°
ACCESS-ESM1-5	Commonwealth Scientific and Industrial Research Organisation, Australia	r1i1p1f1	1.875°×1.2143°
BCC-CSM2-MR	Beijing Climate Center and China Meteorological Administration, China	r1i1p1f1	1.125°×1.125°
CanESM5	Canadian Centre for Climate Modeling and Analysis, Canada	r1i1p1f1	2.8125°×2.8125°
CNRM-CM6-1	National Centre for Meteorological Research and European Centre for Research and Advanced Training in Scientific Computation, France	r1i1p1f2	1.40625°×1.40625°
CNRM-ESM2-1	National Centre for Meteorological Research and European Centre for Research and Advanced Training in Scientific Computation, France	r1i1p1f2	1.40625°×1.40625°
EC-Earth3-Veg	EC-Earth consortium, Europe	r1i1p1f1	0.703125°×0.703125°
FGOALS-g3	LASG, Institute of Atmospheric Physics, Chinese Academy of Sciences, China	r1i1p1f1	2°×2.25°
GFDL-ESM4	National Oceanic and Atmospheric Administration Geophysical Fluid Dynamics Laboratory, United States	r1i1p1f1	1.25°×1°
HadGEM3-GC31-LL	Met Office Hadley Centre, UK	r1i1p1f3	1.875°×1.25°
INM-CM4-8	Institute for Numerical Mathematics, Russia	r1i1p1f1	2°×1.5°
INM-CM5-0	Institute for Numerical Mathematics, Russia	r1i1p1f1	2°×1.5°
IPSL-CM6A-LR	Institute Pierre Simon Laplace, France	r1i1p1f1	2.5°×1.25874°
MIROC6	University of Tokyo, National Institute for Environmental Studies and Japan Agency for Marine-Earth Science and Technology, Japan	r1i1p1f1	1.40625°×1.40625°
MIROC-ES2L	University of Tokyo, National Institute for Environmental Studies and Japan Agency for Marine-Earth Science and Technology, Japan	r1i1p1f2	2.8125°×2.8125°
MPI-ESM1-2-HR	Max Planck Institute for Meteorology, Germany	r1i1p1f1	0.9375°×0.9375°
MPI-ESM1-2-LR	Max Planck Institute for Meteorology, Germany	r1i1p1f1	1.875°×1.875°
MRI-ESM2-0	Meteorological Research Institute, Japan	r1i1p1f1	1.125°×1.125°
NESM3	Nanjing University of Information Science and Technology, China	r1i1p1f1	1.875°×1.875°
NorESM2-LM	University of Bergen, Norway	r1i1p1f1	2.5°×1.875°
NorESM2-MM	Norwegian Climate Centre, Norway	r1i1p1f1	1.25°×0.9375°
UKESM1-0-LL	Met Office Hadley Centre, UK	r1i1p1f2	1.875°×1.25°

Supplementary Table 5. Annual mean values of WATCH Forcing Data (WFD)-based precipitation and temperature, the ensemble of 22 native and bias-corrected CMIP6 model output for the calibration (1980-2000) and validation (2001-2014) period. Note that the precipitation in Indus is adjusted by multiplying the WFD by 1.61.

		Yellow	Yangtze	Mekong	Salween	Brahmaputra	Ganges	Indus
Precipitation (mm, Calibration/Validation)	WFD	518/515	373/405	615/593	643/642	486/558	1551/1371	592/543
	Native	1082/1083	671/693	1458/1479	1499/1523	1390/1410	1184/1230	969/986
	Bias-corrected	513/516	370/384	603/611	630/642	497/507	1531/1597	549/562
Temperature (°C, Calibration/Validation)	WFD	-1.2/-0.8	-5.3/-4.7	-0.7/-0.2	-0.1/0.5	-0.4/0.5	5.4/6.2	-0.2/0.6
	Native	-3.7/-3.0	-6.5/-5.9	-0.8/-0.3	0.4/0.9	1.7/2.3	17.5/17.9	2.1/2.8
	Bias-corrected	-1.3/-0.6	-5.4/-4.8	-0.6/-0.1	-0.2/0.3	-0.3/0.2	6.5/6.9	0.0/0.7

Supplementary Table 6. The end year of each GCM under SSP245 and SSP585 to obtain specific temperature increases.

Models	1.5°C		2°C		3°C	
	SSP245	SSP585	SSP245	SSP585	SSP245	SSP585
ACCESS-CM2	2031	2029	2041	2040	2062	2058
ACCESS-ESM1-5	2031	2031	2046	2044	2074	2062
BCC-CSM2-MR	2035	2032	2049	2043	2094	2064
CanESM5	2027	2027	2036	2035	2056	2050
CNRM-CM6-1	2039	2037	2054	2048	2084	2066
CNRM-ESM2-1	2035	2034	2055	2048	2084	2069
EC-Earth3-Veg	2035	2032	2052	2044	2080	2064
FGOALS-g3	2039	2037	2061	2055	-	2078
GFDL-ESM4	2035	2038	2057	2053	-	2075
HadGEM3-GC31-LL	2025	2025	2034	2033	2054	2049
INM-CM4-8	2042	2038	2060	2051	-	2073
INM-CM5-0	2038	2035	2062	2048	-	2072
IPSL-CM6A-LR	2032	2032	2044	2044	2068	2061
MIROC6	2040	2038	2056	2052	-	2073
MIROC-ES2L	2036	2036	2053	2047	2093	2068
MPI-ESM1-2-HR	2036	2037	2058	2053	-	2076
MPI-ESM1-2-LR	2043	2041	2060	2056	-	2077
MRI-ESM2-0	2032	2029	2046	2040	2076	2061
NESM3	2028	2027	2041	2037	2071	2056
NorESM2-LM	2043	2041	2068	2058	-	2080
NorESM2-MM	2042	2039	2061	2053	-	2078
UKESM1-0-LL	2027	2027	2036	2035	2052	2048
Average	2035	2034	2051	2046	2073	2066

Supplementary Table 7. Details of the THREW model parameters.

Symbol	Unit	Description	Range
kv		Fraction of potential transpiration rate over potential evaporation	0.001~0.8
nt	-	Manning roughness coefficient of hillslope	0.0001~0.2
GaIFL	-	Spatial heterogeneous coefficient of infiltration capacity	0.001~0.7
GaEFL	-	Spatial heterogeneous coefficient of exfiltration capacity	0.0001~0.7
GaETL	-	Spatial heterogeneous coefficient of evapotranspiration capacity	0.0001~0.7
WM		Tension water storage capacity	0.1~10
B	-	Shape coefficient for calculating the saturation excess streamflow area	0.001~1
Gatr		Coefficient representing spatial heterogeneity of exchange term between t-zone and r-zone	0.001~10
KKA	-	Coefficient for calculating subsurface flow in $R_g=KKD\cdot S\cdot K_S^S(\frac{y^S}{Z})^{KKA}$, When S is the topographic slope, y^S is the depth of zone, Z is the total soil depth	0.01~6
KKD	-	See describe for KKA	0.0001~0.5
DDF_S	mm °C^{-1} day^{-1}	Degree day factor for snow	0.001~10
C1	-	Coefficient for calculating the runoff concentration process using Muskingum method: $O_2=C_1\cdot I_1+C_2\cdot I_2+C_3\cdot O_1+C_4\cdot Q_{lat}$$O_2=C_1\cdot I_1+C_2\cdot I_2+C_3\cdot O_1+C_4\cdot Q_{lat}$, where I_1 and O_1 is the inflow and outflow at the prior step, I_2 and O_2 is the inflow and outflow at current step, Q_{lat} is the lateral flow of the river channel, $C_3=1-C_1-C_2$, $C_4=C_1+C_2$	0.0001~1
C2	-	See description for C_1	0.0001~1
LL	-	Coefficient used in snow depletion curve for calculating snow cover area in $SCA=A\cdot(\frac{SWE}{WMAX})^{LL}$	0.0001~1
WMAX	mm	See description for LL	0.001~10
A	-	See description for LL	0.001~10
m_g	-	Parameters in glacier volume-area scaling relation $A_g=(\frac{V_g}{m_g})^{ng}$	0.001~10
n_g	-	See description for mg	0.001~10
DDF_G	mm °C^{-1} day^{-1}	Degree day factor for glacier	0.001~10

Supplementary Table 8. The criteria used to evaluate the model performance in this study.

Name	Formula	Range	Ideal value
Nash Sutcliffe efficiency coefficient (NSE)	$NSE = 1 - \frac{\sum_{t=1}^{n}(Q_{s,t} - Q_{o,t})^2}{\sum_{t=1}^{n}(Q_{o,t} - \overline{Q_o})^2}$	(-∞,1)	1
Correlation coefficient (CC)	$CC = \frac{\sum_{t=1}^{n}\left(Q_{s,t} - \overline{Q_s}\right) \cdot (Q_{o,t} - \overline{Q_o})}{\sqrt{\sum_{t=1}^{n}(Q_{s,t} - \overline{Q_s})^2 \cdot \sum_{t=1}^{n}(Q_{o,t} - \overline{Q_o})^2}}$	(-1,1)	1
Percent bias (PBIAS)	$PBIAS = \frac{\sum_{t=1}^{n}(Q_{s,t} - Q_{o,t})}{\sum_{t=1}^{n} Q_{o,t}} \cdot 100$	(-∞,+∞)	0

Note that: $Q_{o,t}$ and $Q_{s,t}$ are the sequence of observed and simulated variables, respectively; $\overline{Q_o}$ and $\overline{Q_s}$ are mean values of the observed and simulated variables, respectively; n denotes the total number of days in the calibration period.

Supplementary Table 9. The optimal value of the THREW model parameters.

Parameter	Yellow	Yangtze	Mekong	Salween	Brahmaputra	Ganges		Indus
						Karnali	Gandaki	
kv	0.492	0.702	0.56	0.771	0.780	0.101	0.655	0.611
nt	0.107	0.182	0.152	0.147	0.051	0.001	0.085	0.005
GaIFL	0.142	0.665	0.081	0.305	0.644	0.111	0.57	0.628
GaEFL	0.341	0.537	0.508	0.214	0.550	0.447	0.623	0.483
GaETL	0.606	0.363	0.302	0.59	0.497	0.115	0.7	0.335
WM	4.203	7.38	4.034	7.445	2.492	9.638	8.7	4.707
B	0.202	0.83	0.282	0.924	0.224	0.015	0.079	0.011
Gatr	0.057	0.055	1.169	1.942	7.736	7.195	8.364	8.416
KKA	4.942	5.299	3.78	1.812	5.603	5.479	5.459	1.109
KKD	0.008	0.005	0.099	0.002	0.190	0.245	0.435	0.087
DDF_S	9.743	9.106	6.533	5.035	5.683	3.107	8.534	4.938
C1+C2	0.604	0.729	0.266	0.912	0.084	0.636	0.164	0.594
C1/(C1+C2)	0.308	0.623	0.812	0.439	0.064	0.488	0.047	0.426
LL	0.587	0.514	0.714	0.737	0.368	0.097	0.924	0.172
WMAX	4.06	2.69	3.995	1.798	1.468	1.457	4.803	1.317
A	4.096	2.63	5.041	4.228	1.014	0.778	8.599	1.089
m_g	2.796	1.932	0.725	0.788	1.734	0.713	0.698	4.972
n_g	1.326	1.324	1.388	1.29	1.402	1.55	1.396	1.200
DDF_G	4.540	5.100	5.298	2.454	2.768	1.658	4.619	3.729

Supplementary Table 10. Comparison of the glacier mass balance between this study and results extracted from two other different datasets for the Brahmaputra and Indus.

No. of REW	Brahmaputra				Indus			
	WGMS (m/yr)	Hugonnet et al. (2021) (m/yr)	Results from this study (m/yr)	Period	WGMS (m/yr)	Hugonnet et al. (2021) (m/yr)	Results from this study (m/yr)	Period
1	-0.025	-0.831	-0.079	2000-2010	0.098	0.116	-0.128	2000-2008
2	-0.954	-0.704	-0.397	2000-2010	-0.178	0.159	-0.185	2000-2008
3					-0.551	0.178	-0.133	2000-2008
4					-0.174	-0.084	-0.705	2000-2008
5					-0.037	-0.067	-0.212	2000-2010
6					0.35	0.02	-0.496	2000-2010
7					-0.145		-0.346	1999-2007
8					-0.554	-0.273	-1.033	2000-2011
9					-0.775	-0.401	-0.549	2000-2011
Average	-0.489	-0.767	-0.238		-0.218	-0.044	-0.421	

Supplementary Table 11. Sensitivity assessment of future runoff change to different parameters for Indus river.

Different scenario		Future runoff change compared to baseline period		
		1.5 °C	2.0 °C	3.0°C
Baseline	δ = 1.60<br>DDF_G = 3.729	-2.46%	-1.85%	1.45%
Annual glacier change rate (δ)	The upper bound (1.71)	-2.59%	-2.09%	1.09%
	The lower bound (1.01)	-1.83%	-0.62%	3.28%
	+5% (1.68)	-2.55%	-2.03%	1.19%
	-5% (1.52)	-2.37%	-1.68%	1.71%
Degree-day factors (DDF_G)	The upper bound (4.102)	-3.03%	-2.69%	0.24%
	The lower bound (3.356)	-1.88%	-0.98%	2.74%
	+5% (3.915)	-2.74%	-2.27%	0.83%
	-5% (3.543)	-2.17%	-1.42%	2.09%

Supplementary References

1. van Pelt SC, Kabat P, ter Maat HW, van den Hurk BJJM, Weerts AH. Discharge simulations performed with a hydrological model using bias corrected regional climate model input. *Hydrol. Earth Syst. Sci* **13**,2387-2397 (2009).

2. Parajka J, Bloschl G. The value of MODIS snow cover data in validating and calibrating conceptual hydrologic models. *J. Hydrol* **358**,240-258 (2008).

3. He ZH, Parajka J, Tian FQ, Bloschl G. Estimating degree-day factors from MODIS for snowmelt runoff modeling. *Hydrol. Earth Syst. Sci* **18**,4773-4789 (2014).

4. Nan Y, Tian LD, He ZH, Tian FQ, Shao LL. The value of water isotope data on improving process understanding in a glacierized catchment on the Tibetan Plateau. *Hydrol. Earth Syst. Sci* **25**,3653-3673 (2021).

5. Luo Y, Arnold J, Liu SY, Wang XY, Chen X. Inclusion of glacier processes for distributed hydrological modeling at basin scale with application to a watershed in Tianshan Mountains, northwest China. *J. Hydrol* **477**,72-85 (2013).

6. Hock R, Jansson P, Braun LN. Modelling the response of mountain glacier discharge to climate warming. In: *Global change and mountain regions: An Overview of Current Knowledge*. (Springer, Dordrecht, 2005).

7. Chen J, Ohmura A. Estimation of Alpine glacier water resources and their change since the 1870s. *IAHS publ* **193**,127-135 (1990).

8. Shi Y, Liu C, Kang E. The glacier inventory of China. *Ann. Glaciol* **50**,1-4 (2009).

9. RGI Consortium. Randolph Glacier inventory–A dataset of Global glacier outlines: Version 6.0: Technical report, Global land ice measurements from space. https://doi.org/10.7265/N5-RGI-60. (2017)

10. Bolch T, *et al*. Status and change of the cryosphere in the extended Hindu Kush Himalaya region. In: *The Hindu Kush Himalaya Assessment*. (Springer International Publishing, Cham, Switzerland, 2019).

11. Huang L, Li Z, Zhou JM, Zhang P. An automatic method for clean glacier and nonseasonal snow area change estimation in High Mountain Asia from 1990 to 2018. *Remote Sens. Environ* **258**,112376 (2021).

12. Ninglian W, Tandong Y, Baiqing X, An'an C, Weicai W. Spatiotemporal pattern, trend, and influence of glacier change in Tibetan Plateau and surroundings under global warming. *Bulletin of Chinese Academy of Sciences (Chinese Version)* **34**,1220-1232 (2019).

13. Millan R, Mouginot J, Rabatel A, Morlighem M. Ice velocity and thickness of the world's glaciers. *Nat. Geosci* **15**,124-129 (2022).

14. Frey H, *et al*. Estimating the volume of glaciers in the Himalayan–Karakoram region using different methods. *The Cryosphere* **8**,2313-2333 (2014).

15. Chen XN, *et al*. Developing a composite daily snow cover extent record over the Tibetan Plateau from 1981 to 2016 using multisource data. *Remote Sens. Environ* **215**,284-299 (2018).

16. Hadley Centre for Climate Prediction and Research. WATer and global CHange (WATCH) Forcing Data (WFD) - 20th Century. https://rda.ucar.edu/datasets/. (2018)

17. Farr TG, *et al*. The shuttle radar topography mission. *Rev. Geophys* **45** (2007).

18. Tadono T, *et al*. Generation of the 30 M-Mesh Global Digital Surface Model by Alos Prism. *Int Arch Photogramm* **41**,157-162 (2016).

19. Yamazaki D, Ikeshima D, Sosa J, Bates PD, Allen GH, Pavelsky TM. MERIT Hydro: A High-Resolution Global Hydrography Map Based on Latest Topography Dataset. *Water Resour. Res* **55**,5053-5073 (2019).

20. Vermote E. NOAA Climate Data Record (CDR) of AVHRR Normalized Difference Vegetation Index (NDVI), Version 5. https://www.ncei.noaa.gov/products/climate-data-records/normalized-difference-vegetation-index (2019).

21. Vermote E. NOAA Climate Data Record (CDR) of AVHRR Leaf Area Index (LAI) and Fraction of Absorbed Photosynthetically Active Radiation (FAPAR), Version 5. https://www.ncei.noaa.gov/products/climate-data-records/leaf-area-index-and-fapar. (2019)

22. Vermote E, *et al*. NOAA Climate Data Record (CDR) of AVHRR Surface Reflectance, Version 5. http://dx.doi.org/10.7289/V5TM782M. (2019)

23. Guo WQ, *et al*. The second Chinese glacier inventory: data, methods and results. *J Glaciol* **61**,357-372 (2015).

24. WGMS. Fluctuations of Glaciers Database. http://dx.doi.org/10.5904/wgms-fog-2021-05 (2021).

25. Hugonnet R, *et al*. Accelerated global glacier mass loss in the early twenty-first century. *Nature* **592**,726-731 (2021).

26. Dai Y, *et al*. A Global High‐Resolution Data Set of Soil Hydraulic and Thermal Properties for Land Surface Modeling. *J. Adv. Model. Earth Syst* **11**,2996-3023 (2019).
