# Supplementary material for: Non-monotonic changes in Asian Water Towers’ streamflow at increasing warming levels
Source: Nat Commun. 2023 Mar 1;14:1176. doi: 10.1038/s41467-023-36804-6 (PMC9977870; doi:10.1038/s41467-023-36804-6)
Supplement: Supplementary file 3 — Description of Additional Supplementary Files [file 41467_2023_36804_MOESM3_ESM.pdf]

Description of Additional Supplementary Files:

Supplementary Dataset 1: Results of this study. Each file contains the results of annual and seasonal precipitation, runoff, evapotranspiration change, annual snow cover and glacier area change of 22 CMIP6 GCM models under different scenarios. And the averaged annual and seasonal contribution of rainfall runoff, snowmelt runoff, and glacier runoff of 22 CMIP6 GCM models for different scenarios.
